# Medical appointments and provision of medical care during the COVID-19 pandemic in Mainz, Germany

**DOI:** 10.1371/journal.pone.0280292

**Published:** 2023-01-12

**Authors:** Markus Schepers, Irene Schmidtmann, Daniela Zahn, Philipp Wild, Manfred Beutel, Alexander K. Schuster, Thomas Münzel, Karl J. Lackner, Katharina Geschke, Jörn Schattenberg, Rieke Baumkötter, Simge Yilmaz, Daniel Wollschläger

**Affiliations:** 1 Institute of Medical Biostatistics, Epidemiology and Informatics (IMBEI), University Medical Center, Johannes Gutenberg University, Mainz, Germany; 2 Preventive Cardiology and Preventive Medicine, Center for Cardiology, University Medical Center of the Johannes Gutenberg University, Mainz, Germany; 3 Clinic and Polyclinic for Psychosomatic Medicine and Psychotherapy, Mainz, Germany; 4 Department of Ophthalmology, University Medical Center Mainz, Mainz, Germany; 5 Institute for Clinical Chemistry, Central Laboratory, Mainz, Germany; 6 Department of Psychiatry and Psychotherapy, University Medical Center, Johannes Gutenberg University, Mainz, Germany; 7 Schwerpunkt Metabolische Lebererkrankungen I. Medizinische Klinik und Poliklinik Universitätsmedizin Mainz, Mainz, Germany; JSMU: Jinnah Sindh Medical University, PAKISTAN

## Abstract

Previous evidence suggested that non-COVID-19-related medical care was reduced during the first wave of the COVID-19 pandemic, but it remained unclear whether or to which extent this effect lasted beyond the first wave, or existed in a longer time frame. Here, we consider questionnaire data of the Gutenberg-COVID-19 study together with pre-pandemic baseline data of the Gutenberg Health Study concerning the region around Mainz, Germany, to study the effects of the pandemic on the provision of medical care until April 2021. We observed that the proportion of cancelled medical appointments was low and that the fraction of participants with a medical appointment as an indicator for the number of appointments being made was in line with pre-pandemic levels. Appointments were more likely cancelled by the patient (rather than the provider), and more likely cancelled by medical specialists such as dentists or ophthalmologists (rather than GPs). In conclusion, we found some evidence that, at least with regard to realized appointments, the medical system and the provision of medical care were not harmed by the COVID-19 pandemic on a longer time scale.

## Background

The global COVID-19 pandemic has had a wide range of direct and indirect effects. Due to re-allocation of medical capacity to COVID-19 related diseases, fewer resources were available for other health care services. Furthermore, fear of infection might have reduced the willingness to see a physician, thereby causing missed medical appointments. According to current evidence from different disciplines and regions, missed medical appointments substantially increased during the first wave of the COVID-19 pandemic in early 2020 [1]. For example, Schäfer et al. observed a substantial reduction in the number of general practitioner (GP) consultations around Hamburg during the first wave of COVID-19 from April to July 2020 [2]. Many studies considered cardiovascular disease and death, giving warnings of later collateral damage due to a substantial decrease in cardiovascular hospital admissions in the UK in early 2020 [3] and excess acute cardiovascular mortality not attributable to COVID-19 [4]. Regarding cancer, there has been evidence that modestly delaying appointments by 3 to 6 months has a relevant impact on survival [5]. It has also been found that the reduced use of non-COVID-19-related medical appointments lasted beyond lockdown easing [6]. Missed medical appointments even affected routine vaccination appointments [7].

Pinggera et al. reported a noticeable reduction in the number of appointments at their neurosurgical outpatient clinic in Tyrol during the first wave of COVID-19. Their questionnaire survey indicated that these effects were not due to patients’ fear of infection, but rather due to regulatory measures [8]. An early study in March 2020 discovered that dentists around the world were anxious of getting infected due to their exposition to aerosol and droplets splashing out of patients’ oral cavity [9].

Apart from the known fear and reduction of medical care at the beginning of the COVID-19 outbreak it remained unclear how the provision of medical care was affected by the COVID-19 pandemic beyond the first wave. Therefore, we analyzed the unknown long-term of effect of COVID-19 on the medical system with relevant data from the Gutenberg COVID-19 study which is based on a large prospective representative cohort with pre-pandemic baseline data. The overall study question was: what have been the effects of the COVID-19 pandemic on provision of medical care, in particular regarding missed (or postponed) medical appointments, in the study region from February 2020 to April 2021? How do direct effects, e.g. due to provider, compare to indirect effects, e.g. due to fears of the patients? The significance of the study is based on a large sample and on that the study addresses an important public health issue. The objective of the study is a scientific assessment of the health care system, building on previous findings, but looking at a longer time scale.

## Materials and methods

Our study is based on data collected in the Gutenberg COVID-19 Study (GCS), and data of the Gutenberg Health Study (GHS), a longitudinal study with over 15 000 participants running since 2007. Around 80% of GCS-participants were recruited from the GHS, the remainder were newly included younger participants (aged between 25–44 years). The total random sample was stratified by age (25–88 years), gender (m/f) and place of residence (city/county). The GCS-data was obtained via assessments conducted at two time points at intervals of 4 months, which included a test for acute SARS-CoV-2 infection via throat swab and further biomaterial collection as well as a computer-assisted personal interview and a questionnaire to obtain detailed information on medical risk factors, pre-existing conditions, medication, socioeconomic burden of the COVID-19 pandemic, lifestyle, medical care use, pandemic-related behavior and psychosocial variables). In addition, a voluntary smartphone monitoring lasted for at most 8 months [10–12].

The study protocol and study documents were approved by the local ethics committee of the Medical Chamber of Rhineland-Palatinate, Germany (reference no. 837.020.07; original vote: 22.3.2007, latest update: 20.10.2015). According to the tenets of the Declaration of Helsinki, written informed consent was obtained from all participants prior to entering the study.

Written informed consent from GCS study participants does not allow public access to the data. Access to the data in the local database is possible at any time upon request according to the ethics vote. This concept was developed with the local data protection officer and the ethics committee (local ethics committee of the Rhineland-Palatinate Medical Association, Germany). Interested scientists can make their requests to the Gutenberg COVID-19 Study Steering Committee (e-mail: info-gcs@unimedizin-mainz.de).

We performed a complete case analysis on the set of GCS-participants with respect to all variables included in this study. We considered demographic data (age, sex, smoking), baseline health (obesity, cancer, diabetes, stroke) and information regarding health care services for the time period from the beginning of the COVID-19 pandemic in Germany in February 2020 until the third wave in April 2021. Additionally, we then studied the corresponding data on health care services in the GHS-cohort (cancellation of appointments for all available disciplines, such as GP, and types, such as screening) from 2018–2021 for the comparison with pre-pandemic times. We computed descriptive statistics (mean, standard deviation, min, max, absolute and relative frequencies where appropriate). We calculated Pearson’s chi-square test of independence for the variables medical sub-discipline and status of screening appointment and for the variables medical sub-discipline and status of hospital appointment. Here, medical sub-discipline is a categorical variable with 14 possibilities and appointment status is a categorical variable with three possibilities: realized, cancelled by provider and cancelled by patient. We put a particular emphasis on GPs, dentists and ophthalmologists because these are the most commonly used disciplines or have a particularly short face-to-face distance between patient and physician, respectively. We computed a logistic regression for the endpoint that all scheduled appointments of a participant took place. Covariates pre-selected on theoretical grounds were age, sex, BMI, diabetes, stroke, life-time smoker and cancer. We report adjusted odds ratios and their 95% confidence intervals. The data was analyzed using R Version 4.0.2 [13].

## Results

Among the 10250 participants included in the data set there are 9820 participants with complete information in all relevant variables (corresponding to 430 participants (4.2%) with missing values). This study sample had an average age of 56.1 years with a standard deviation of 15.7 and included a fraction of 50.8% females. There were 2299 participants with obesity (22.4%) and 982 participants with cancer (9.6%). 927 participants had diabetes (9%) and 217 participants had a stroke (2.1%).

7029 (72.8%) participants had at least one medical appointment.

Among all participants who had a medical appointment, there were 950 (13.5%) participants with at least one appointment that they cancelled themselves and 605 (8.6%) participants with at least one appointment that was cancelled by the provider. Taken together, there were 1435 (20.4%) participants with at least one appointment which was cancelled (less than the sum of own and provider cancellations as there were participants who had both).

The medical sub-discipline (with 14 possibilities) and the cancellation of screening appointments were not independent (Pearson’s chi-square test chi^2(df = 26,n = 9820) = 223.1, p <0.001). Likewise, regarding hospitalizations, the medical sub-discipline (with 12 possibilities, as GP and psychiatry had missing values) and cancellation (with three possibilities) were not independent (Pearson’s chi-square test chi^2(df = 22,n = 9820) = 135.6, p <0.001).

**Fig 1:**
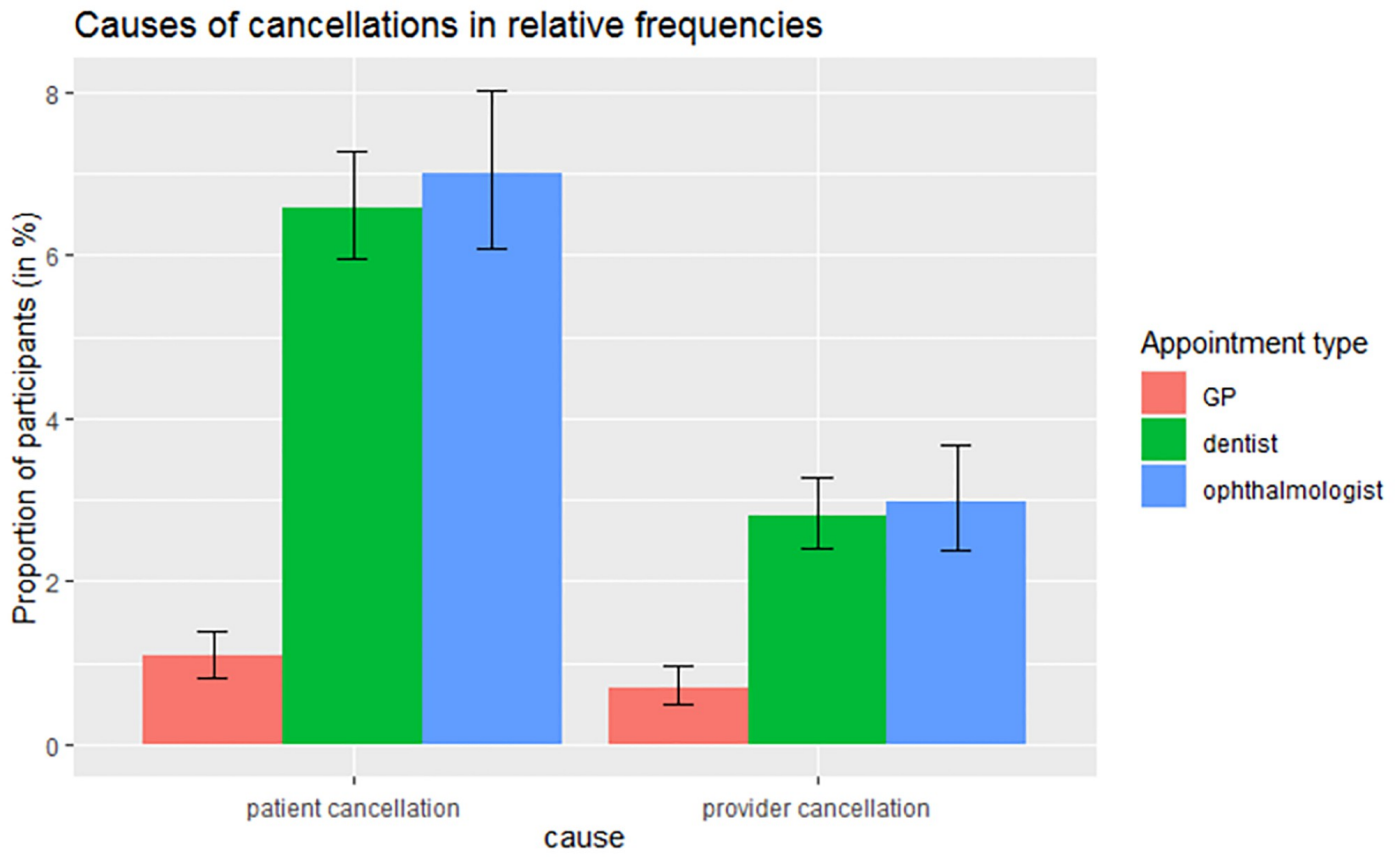
Comparison of relative frequency of cancelled appointments among GPs, dentists and ophthalmologists (each percentage is with respect to the number of participants who had made any appointment in the respective category), showing Clopper-Pearson confidence intervals for binomial proportions.

Table 1 and Fig 1 show that most appointments were realized. Appointments with medical specialists like dentists and ophthalmologists had a higher relative frequency of being cancelled than GP appointments.

**Table 1:** Comparison of appointment status for GP, dentist and ophthalmologist.

	GP N (N/T * 100%)	Dentist N (N/T * 100%)	Ophthalmologist N (N/T * 100%)
Participants with at least one appointment, All realized	5199 (98.24%)	5163 (90.67%)	2526 (90.25%)
Participants with at least one appointment, At least one cancelled by participant	57 (1.08%)	375 (6.59%)	196 (7.00%)
Participants with at least one appointment, At least one cancelled by provider	37 (0.70%)	160 (2.81%)	83 (2.97%)
Participants with at least one appointment T (total)	5292 (100%)	5694 (100%)	2799 (100%)

There were 118 participants with information on the cancellation of cancer-check-up appointments from 982 participants with cancer. Of these, for 114 participants, the cancer-check-up appointment was not cancelled, for 2 participants, the cancer-check-up appointment was postponed, for 2 participants, the cancer-check-up appointment was cancelled.

Among the 982 participants with cancer 84 participants provided information on the cancellation of cancer-chemotherapy appointments. Of these, for 80 participants, the cancer-chemotherapy appointment was not cancelled, for 2 participants, the cancer-chemotherapy appointment was postponed, for 2 participants, the cancer-chemotherapy appointment was postponed multiple times.

There were 8834 participants with data on the change of medication. Of these, 81 participants (0.9%) took medication which was changed during the COVID-19 pandemic, while 6233 participants (70.6%) took medication but it was not changed. The remaining 2520 participants (28.5%) did not take any medication.

**Fig 2:**
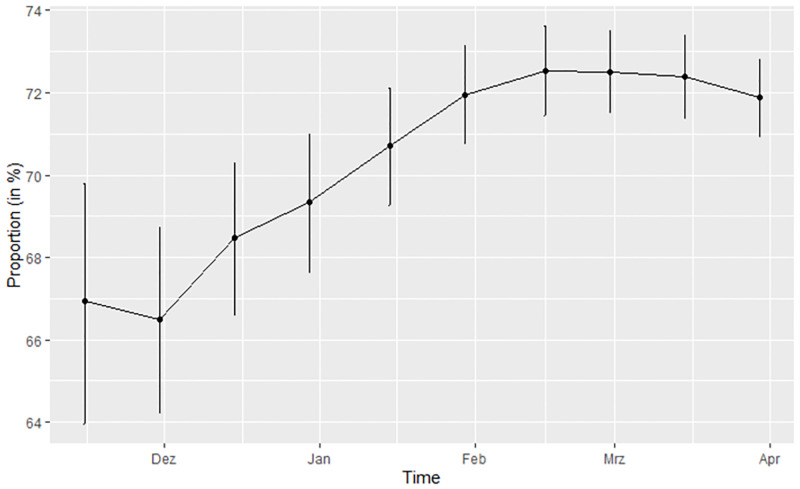
(Cumulative) relative frequency of medical appointment over time, with Clopper-Pearson confidence intervals for binomial proportions.

Regarding the temporal evolution of appointments (see Fig 2), the relative frequency of participants with medical appointment was lowest at the beginning of the second wave with around 66%. During the peak of the wave during January and February 2021, the relative frequency of participants with medical appointment increased to a cumulative relative frequency of around 73%.

In logistic regression for the endpoint that all scheduled appointments took place, women had lower odds that all their appointments took place (odds ratio 0.78 (95% CI 0.69–0.88)). For participants with stroke (odds ratio 0.81 (0.56–1.17)), diabetes (odds ratio 1.02 (0.84–1.24)), life-time smokers (odds ratio 0.92 (0.82–1.03)) and cancer patients (odds ratio 1.01 (0.86–1.18)) no (significant) difference was detected.

Regarding the GHS baseline data, the fraction of participants with a dentist’s appointment in the previous 12 months was 0.76 and 0.79 in 2018 and 2019 respectively and 0.78, 0.79 and 0.80 for 2020, 2021 and 2022 respectively. The corresponding fraction of participants with a GP appointment in the previous 12 months was 0.78 and 0.80 in 2018 and 2019 respectively and 0.79, 0.77 and 0.79 for 2020, 2021 and 2022 respectively, thus showing little evidence for a decline of medical appointments during the pandemic (even though some of these proportions have a statistically significant difference at the 5% level due to the large sample size, the differences between the proportions do not appear to be relevant from a practical point of view). However, for ophthalmologist’s appointments there appears to be a slight drop: a fraction of 0.50 and 0.40 of participants reported an appointment with an ophthalmologist in the previous 12 months in 2018 and 2019 respectively, whereas the corresponding fraction was 0.36, 0.32 and 0.33 for 2020, 2021 and 2022 respectively.

## Discussion

Looking at the GCS data covering the time period from February 2020 to April 2021, we observed that among the 70% of study participants with a medical appointment in that period, around 20% had an appointment that did not take place (being cancelled by themselves or the provider). However, this relatively high percentage is with respect to study participants, i.e. aggregated over all their appointments, rather than with respect to single appointments. A substantial fraction of participants had multiple appointments, in different disciplines. Indeed, a closer look reveals that, for instance, less than 2% of participants with a GP appointment reported that such an appointment did not take place. This suggests that the fraction of missed medical appointments that were reported (in the study period from February 2020 to April 2021) is consistent with the pre-pandemic situation: In a pre-pandemic context, a German study found an average of 6% of appointments that were cancelled at short notice and another 6% of no-shows [14].

That the fraction of missed or cancelled appointments during the pandemic was comparable to the pre-pandemic years also does not seem to be due to fewer appointments being made in the first place: Based on GHS data, i.e. concerning the region under study with a major overlap with the GCS study participants, the fraction of participants with a medical appointment remained relatively constant during the COVID-19 pandemic. Our results seem to diverge from prior studies mentioned in the introduction, where substantial decreases in medical care during the first wave of COVID-19 were reported. As a potential explanation we suspect that we are looking at a longer time-frame, not only the peak of (one/the first) wave. Indeed, the longer time period for a relatively large cohort is a strength, or at least a notable motivation, of our study. During the course of the COVID-19 pandemic, the medical system appears to have adapted successfully to the new situation.

Note that there are several limitations of our study. First of all, our analysis is always based on participants and medical disciplines due to a lack of more fine-grained data on the single-appointment level, i.e. all relevant data on every single appointment (by a study participant in the study period) that has ever been planned. We assume (due to the similarity and causal link between the two perspectives) that our results are still reliable and realistic. Furthermore, the questionnaire for the GCS was directly developed for this study as there was no template available (however note that the GHS questionnaire is using standardized questions that have also been used in other cohort studies).

Even though the fraction of missed or cancelled appointments turned out to be low, it is an important quantity to consider because it might have drastic effects for the affected individuals (e.g. angina pectoris or heart attacks, which we cannot cover in this detail with our data). We did not consider changes in stationary medical care. As a final limitation, note that individuals may feel a higher psychological burden than what is reflected by appointment numbers (e.g. anxiety during appointments with precautionary measures).

Future studies could look at a more fine-grained, i.e. single appointment-based level, or explicitly focus on patients with a large number of appointments (during a single year). It would also be interesting to zoom into a particular discipline, e.g. dentistry, and compare the provision of medical appointments (realized, postponed or cancelled) between dentists that implemented preventive hygiene measures and those that failed to do so. This would allow to check whether the updated hygiene guidelines are effective at maintaining normal provision of medical care.

To conclude, we observed a negligible, if any, overall effect or collateral damage of the COVID-19 pandemic (from February 2020 to April 2021) on the realization of medical appointments in the region under study.
